# The effect and safety of postmenopausal hormone therapy and selective estrogen receptor modulators on kidney outcomes in women: a protocol for systematic review and meta-analysis

**DOI:** 10.1186/s13643-017-0519-2

**Published:** 2017-07-07

**Authors:** Sandra M. Dumanski, Sharanya Ramesh, Matthew T. James, Amy Metcalfe, Kara Nerenberg, Ellen W. Seely, Helen Lee Robertson, Sofia B. Ahmed

**Affiliations:** 1 Division of Nephrology, Department of Medicine, University of Saskatchewan, 103 Hospital Drive, Saskatoon, SK S7N 0W8 Canada; 2 Cumming School of Medicine, University of Calgary, 3330 Hospital Drive NW, Calgary, AB T2N 4N1 Canada; 3 Department of Medicine, University of Calgary, 3330 Hospital Drive NW, Calgary, Alberta T2N 4N1 Canada; 4 Alberta Kidney Disease Network, 1403 29th St NW, Calgary, AB T2N 2T9 Canada; 5 Department of Obstetrics and Gynecology, University of Calgary, 3330 Hospital Drive NW, Calgary, AB T2N 4N1 Canada; 6 Department of Community Health Sciences, University of Calgary, 3330 Hospital Drive NW, Calgary, AB T2N 4N1 Canada; 7 Division of Endocrinology, Diabetes and Hypertension, Department of Medicine, Brigham and Women’s Hospital, 75 Francis St, Boston, MA 02115 USA; 8 Health Sciences Library, University of Calgary, 3330 Hospital Drive NW, Calgary, AB T2N 4N1 Canada; 9 Libin Cardiovascular Institute of Alberta, 1403 29th St NW, Calgary, AB T2N 2T9 Canada

**Keywords:** Women, Kidney, Hormone therapy, SERM, Albuminuria, Menopause

## Abstract

**Background:**

The prevalence of menopause in women with or at risk of chronic kidney disease is increasing globally. Although international guidelines on menopause recommend the use of postmenopausal hormone therapy with or without selective estrogen receptor modulators for control of vasomotor symptoms, the effects of these treatments on kidney function and albuminuria are unclear. Furthermore, women with chronic kidney disease are at significantly increased risk of venous thromboembolism and malignancy, well-documented adverse effects of postmenopausal hormone therapy. Our study aims to establish the effect of these treatments on kidney function and albuminuria in women, as well as determine the safety of these treatments in the chronic kidney disease population.

**Methods:**

We will conduct a systematic review and meta-analysis addressing the effect and safety of postmenopausal hormone therapy and selective estrogen receptor modulators on kidney outcomes in women. We plan to search for published (MEDLINE, EMBASE, Cochrane Central Register of Controlled Trials (CENTRAL), tables of contents of relevant journals) and unpublished (ongoing studies, conference proceedings) studies in all languages examining the effect of postmenopausal hormone therapy, including selective estrogen receptor modulators, on kidney function and albuminuria, as well as the risk of adverse outcomes of these treatments in women with chronic kidney disease. Two independent investigators will screen identified abstracts and select studies that examine the effect of postmenopausal hormone therapy and selective estrogen receptor modulators on kidney outcomes in the general population or adverse outcomes in the chronic kidney disease population. Data on study population, intervention, outcomes, as well as study quality and risk of bias will be independently extracted from each eligible study. Along with descriptive presentation of data, outcome measures will be presented as meta-analyses using a random effects model. Planned subgroup analyses will be completed, and meta-regression will be performed if significant heterogeneity is noted.

**Discussion:**

By examining the effects of postmenopausal hormone therapy and selective estrogen receptor modulators on kidney function and albuminuria, the results of this systematic review and meta-analysis will inform management of postmenopausal women in the general population. Furthermore, it will evaluate the safety, including the risks of known adverse outcomes of postmenopausal hormone therapy and selective estrogen receptor modulators, in the already vulnerable chronic kidney disease population.

**Systematic review registration:**

PROSPERO CRD42016050651

**Electronic supplementary material:**

The online version of this article (doi:10.1186/s13643-017-0519-2) contains supplementary material, which is available to authorized users.

## Background

### Menopause

The prevalence of menopause, defined as the natural cessation of menses for 1 year [1], is increasing as the world’s population ages [2]. Globally, it is estimated that the number of women with menopause will be greater than one billion by the year 2025 [3]. Menopause is often accompanied by burdensome symptoms, including vasomotor symptoms (VMS), that persist for 7 years or longer in the average woman [4]. Current international guidelines recommend the use of postmenopausal hormone therapy (HT) for management of VMS [1, 5–7]. Estrogen therapy remains the gold standard for postmenopausal HT and is often combined with progestin therapy for women with an intact uterus but may also be combined with a selective estrogen receptor modulator (SERM) [3].

### Menopause and chronic kidney disease

Globally, the prevalence of chronic kidney disease (CKD) is increasing at a rapid rate [8]. Coupled with an aging global population [2], there is significant growth in the number of postmenopausal-aged women with CKD [9–12]. It is estimated that the number of postmenopausal women with end-stage kidney disease (ESKD) is increasing by greater than 4% each year [13]. Compounding this issue, CKD itself is associated with hypothalamic-pituitary-ovarian dysfunction [14, 15], resulting in an earlier onset of menopause, which occurs on average of 5 years earlier than in the general population [16–18]. Despite international guidelines on the use of postmenopausal HT for the general population [1, 5–7], no specific recommendations exist for women with or at risk of CKD.

### Postmenopausal HT and kidney outcomes

The impact of menopause, postmenopausal HT, and SERMs on kidney function in women is unclear: previous meta-analyses have demonstrated conflicting results regarding the influence of sex on the loss of kidney function [19–21]. While an initial meta-analysis suggested that loss of kidney function is more rapid in men than in women [19], a subsequent meta-analysis suggested that the apparent renoprotective benefits of female sex may be limited to premenopausal women [20]. However, a recent meta-analysis of over two million patients showed no difference in CKD progression between the sexes, even after accounting for age as a marker of menopause [21]. One of the reasons behind these discrepant results may lie in the fact that none of these meta-analyses reported on the use of postmenopausal HT or SERMs, which may have potential effects on kidney outcomes. At present, though the complexity of the physiologic and clinical interaction between female sex hormones and kidney function remains poorly understood, animal data report a renoprotective role for estrogen [22–27]. These findings suggest that the estrogen component of postmenopausal HT may play a beneficial role with respect to kidney function, though the effect of postmenopausal HT and SERMs on human kidney function is not yet established.

### Adverse effects of postmenopausal HT

Postmenopausal HT and SERMs are associated with significant adverse effects. Some of the most serious adverse effects of postmenopausal HT in the general population are venous thromboembolism (VTE) and breast, ovarian, and endometrial malignancy [28]. SERMs, though protective in the case of breast cancer [29], are likewise associated with an increased risk of VTE [30]. These adverse effects are of particular concern in women with CKD, given the significantly increased risk of both malignancy and VTE in the setting of CKD [31–33]. As such, it is necessary to investigate whether these adverse effects of postmenopausal HT and SERMs are amplified in women with CKD and to determine the risk-benefit ratio in this population.

### Study objectives

In summary, the number of women of postmenopausal age is increasing exponentially on a global scale [2, 11, 12]. Paralleled by a surge in the prevalence of CKD [8], this has resulted in a rising number in postmenopausal women with or at risk of CKD. The benefits and risks of postmenopausal HT and SERMs on kidney health outcomes in women with and without CKD are poorly understood. We hypothesize that postmenopausal hormone therapy, at least in the non-oral form, is associated with decreased loss of kidney function and lower levels of albuminuria in women. Our study aims to (1) determine the effects of postmenopausal HT, including SERMs, on kidney function and albuminuria in women with or without CKD as well as (2) establish the risk of adverse outcomes of postmenopausal HT, including SERMs, in the CKD population.

## Methods

### Protocol

This systematic review protocol to assess the effect of postmenopausal HT on kidney function and albuminuria in women was developed using the Preferred Reporting Items for Systematic Reviews and Meta-Analyses for Protocols (PRISMA-P) [34] and the Cochrane guidelines for systematic and meta-analysis [35]. An additional file demonstrates this in detail (see Additional file 1: PRISMA-P Checklist). The final protocol was registered with PROSPERO (CRD42016050651) [36].

### Study eligibility

As the purpose of this review is twofold, we will conduct two searches: (1) to examine the effect of postmenopausal HT, including SERMs, on measures of kidney function in women (search 1); (2) to evaluate the risks of adverse effects of postmenopausal HT in women with CKD (search 2). Eligible studies will include randomized controlled trials (RCTs), quasi-RCTs, cohort studies, and case control studies that examine exposure to postmenopausal HT in women with and without CKD. Outcomes in women with and without kidney disease will include kidney function (estimated glomerular filtration rate), albuminuria, progression to ESKD, and hypertension. Outcomes specific to the CKD population will include VTE and breast, ovarian, or endometrial malignancy. The population, exposure, comparator, outcome and study design (PECOD) used to assess study eligibility for this systematic review is presented in Table 1. This search will include all languages, and will be limited to human subjects. Studies published between 1950 and 31 December 2016 will be included.

**Table 1:** PECOD for the systematic review

	PECOD for search #1	PECOD for search #2
Population	Postmenopausal women	Postmenopausal women with chronic kidney disease
Exposure	Hormone therapy and/or selective estrogen receptor modulators	Hormone therapy and/or selective estrogen receptor modulators
Comparator	Placebo or No hormone therapy	Placebo or No hormone therapy
Outcome	1°: Kidney function, Albuminuria	Venous thromboembolism and breast, ovarian or endometrial malignancy
	2°: Progression to end stage kidney disease, hypertension	
Design	RCTs, quasi-RCTs, cohort studies and case control studies	RCTs, quasi-RCTs, cohort studies and case control studies

Description: Detailed description of the PECOD used for Search #1 and Search #2 for this systematic review

### Information sources

An electronic search of MEDLINE (1950–2016), EMBASE (1980–2016), and Cochrane Central Register of Controlled Trials (CENTRAL) will be conducted by a medical librarian. The MEDLINE search will be peer reviewed [37]. Two independent reviewers will review citations for eligibility and reference lists from included articles and relevant reviews, as well as trials registered on the ClinicalTrials.gov website. Abstracts from all major North American, European, Australasian, and British nephrology, endocrinology, gynecology, and menopause meetings (Table 2) will be assessed between 2013 and 2016. Tables of contents from all major nephrology, endocrinology, gynecology, and menopause journals (Table 3) between 2013 and 2016 will be searched. Experts in this field in will be identified through the review process and will be contacted regarding any ongoing or unpublished studies.

**Table 2:** Major scientific meetings

Nephrology	• American Society of Nephrology Kidney Week
	• Canadian Society of Nephrology Annual General Meeting
	• Australian and New Zealand Society of Nephrology Annual Scientific Meeting
	• European Renal Association-European Dialysis and Transplant Association Congress
	• United Kingdom Kidney Week
Gynecology	• American College of Obstetrics and Gynecology Annual General Meeting
	• Society of Obstetricians and Gynaecologists of Canada Annual Clinical and Scientific Meeting • Congress of the European Society of Gynecology
Endocrinology	• American Association of Clinical Endocrinologists Annual Scientific & Clinical Congress
	• Diabetes Canada/Canadian Society of Endocrinology and Metabolism Professional Conference
	• European Congress of Endocrinology
Menopause	• North American Menopause Society Annual Meeting
	• Australasian Menopause Society Congress
	• European Congress on Menopause and Andropause
	• British Menopause Society Annual Conference
	• International Menopause Society World Congress on Menopause

Description: Detailed listing of the scientific meetings in which abstract review will be completed

**Table 3:** Major scientific journals

Nephrology	• *Kidney International*
	• *Journal of the American Society of Nephrology*
	• *Clinical Journal of the American Society of Nephrology*
Gynecology	• *Obstetrics & Gynecology*
	• *American Journal of Obstetrics and Gynecology* • *British Journal of Obstetrics and Gynaecology*
Endocrinology	• *Endocrine Reviews*
	• *Lancet Diabetes & Endocrinology*
	• *Trends in Endocrinology and Metabolism*
Menopause	• *Menopause*
	• *Journal of Women’s Health*
	• *Journal of the British Menopause Society*

Description: detailed listing of the scientific journals in which table of contents will be searched

### Search strategy

Two separate searches will be conducted. The initial search strategies were developed for MEDLINE and were adapted for all other searches.

### Search #1

The purpose of this search is to identify articles that assess the effect of postmenopausal HT on kidney function and albuminuria in postmenopausal women. It will include the exposure and outcomes of the PECOD question in Table 1. The first Boolean search will be completed using the “OR” term to combine MeSH subject headings and/or text words that represent *Postmenopausal HT and SERMs.* The second Boolean search will be completed using the “OR” term to combine exploded MeSH subject headings and/or text words that represent *Albuminuria*. The third Boolean search will be completed using the “OR” term to combine exploded MeSH subject headings and/or text words that represent *Kidney Function*. The fourth Boolean search will be completed using the “OR” term to combine exploded MeSH subject headings and/or text words that represent *Hypertension*. The final search will ensure that each outcome (Kidney Function, Albuminuria, and Hypertension) and its amalgamated terms will be linked using the “OR” operator. The linked outcomes will then be combined using the “AND” operator for the exposure (postmenopausal hormone therapy and SERMs) and its amalgamated terms (Table 4).

**Table 4:** MEDLINE search strategy for Search #1

1.	Hormone replacement therapy/ or estrogen replacement therapy/
2.	Exp Estradiol/tu, th [Therapeutic use, therapy]
3.	Estrogens/tu, th [Therapeutic use, therapy]
4.	Epimestrol/tu, th [Therapeutic use, therapy]
5.	“Estrogens, conjugated (usp)”/tu, th [Therapeutic use, therapy]
6.	“Estrogens, esterified (usp”/tu, th [Therapeutic use, therapy])
7.	Ethinyl estradiol/tu, th [Therapeutic use, therapy]
8.	Mestranol/tu, th [Therapeutic use, therapy]
9.	Exp quinestrol/tu, thy [Therapeutic use, therapy]
10.	Progesterone/tu, th [Therapeutic use, therapy]
11.	Algestone/tu, th [Therapeutic use, therapy]
12.	Hydroxyprogesterones/tu, th [Therapeutic use, therapy]
13.	Exp selective estrogen receptor modulators/tu, th [Therapeutic use, therapy]
14.	Exp Medroxyprogesterone/tu, th [Therapeutic use, therapy]
15.	Exp Progestins/tu, th [Therapeutic use, therapy]
16.	((Hormone or estrogen or oestrogen) adj (replacement or substitution or therap*)).mp.
17.	((Hormone or estrogen or oestrogen) adj (replacement or substitution) adj therap*).mp.
18.	(Selective adj (estrogen or oestrogen) adj receptor adj modulator*).mp.
19.	SERM.mp.
20.	(Medroxyprogesterone adj acetate).mp.
21.	(Estradiol* or oestradiol* or estrogen* or oestrogen* or estriol* or oestriol* or estradiol* or oestradiol*).mp.
22.	(HRT or PHT).ti,kw.
23.	(Progestin* or progesterone* or medroxyprogesterone* or gestagen).mp.
24.	or/1-23
25.	Proteinuria/or exp albuminuria/
26.	(Proteinuria or albuminuria or microalbuminuria or macroalbuminuria or MAU).mp.
27.	(Urinary adj2 (albumin or protein) adj2 excretion).mp.
28.	((Albumin or protein) adj creatinine adj ratio).mp.
29.	((Moderate* or severe*) adj3 increased adj3 (albuminuria or proteinuria)).mp.
30.	or/25-29
31.	Renal insufficiency/ or renal insufficiency, chronic/ or kidney failure, chronic/
32.	Exp glomerular filtration rate/
33.	Exp dialysis/
34.	Exp kidney transplantation/
35.	Renal replacement therapy/ or renal dialysis/ or hemodiafiltration/ or hemodialysis, home/ or peritoneal dialysis/ or peritoneal dialysis, continuous ambulatory/
36.	((Renal or kidney) adj allograft).mp.
37	((Renal or kidney) adj transplant*).mp.
38.	(Predialys* or pre-dialys*).mp.
39.	RRT.ti, kw.
40.	((Renal or kidney) adj replace* adj (therapy or treatment)).mp.
41.	(Peritoneal adj dialys*).mp.
42.	(Hemodiafiltration or hemofiltration or haemodiafiltration or haemofiltration or hemodialys* or haemodialys* or dialys*).mp.
43.	(ESRD or ESKD).ti, kw.
44.	(End adj stage adj (renal or kidney) adj disease*).mp.
45.	((Renal or kidney) adj (insufficien* or failure or disease* or impair*)).mp.
46.	((Renal or kidney) adj function).mp.
47.	(Glomerular adj filtration adj rate*).mp.
48.	(Nephropathy or nephropathies).mp.
49.	(Creatinine adj clearance).mp.
50.	Cystatin.mp.
51.	(CKD or CRI).ti, kw.
52.	(GFR or eGFR).ti, kw.
53.	or/31-52
54.	Hypertension/
55.	Blood pressure/
56.	((Elevate* or high or rais* or heighten* or increas*) adj (“blood pressure” or “diastolic” or “systolic”)).ti, kw.
57.	Hypertensi*.ti, kw.
58.	“Blood pressure”.ti, kw.
59.	or/54-58
60.	(30 or 53 or 59) and 24
61.	Remove duplicates from 60

Description: MEDLINE key word and text word search strategy for Search #1

### Search #2

The purpose of this search is to select articles that assess for adverse effects of postmenopausal HT in the CKD population. It will include the population, exposure, and outcomes of the PECOD question in Table 1. The first Boolean search will be completed using the “OR” term to combine exploded MeSH subject headings and/or text words that represent *Chronic Kidney Disease*. The second Boolean search will be completed using the “OR” term to combine exploded MeSH subject headings and/or text words that represent *Postmenopausal HT and SERMs*. The third Boolean search will be completed using the “OR” term to combine exploded MeSH subject headings and/or text words that represent *Thromboembolic Disease*. The fourth Boolean search will be completed using the “OR” term to combine exploded MeSH subject headings and/or text words that represent *Breast, Ovarian, or Endometrial Malignancy*. The final search will combine the amalgamated terms for each outcome (Thromboembolic Disease and Breast, Ovarian, or Endometrial Malignancy) using the “OR” term. The linked outcomes will then be combined using the “AND” term with the amalgamated terms for the exposure (Postmenopausal Hormone Therapy and SERMs) and the population (Chronic Kidney Disease) (Table 5).

**Table 5:** MEDLINE Search Strategy for Search #2

1.	Hormone replacement therapy/ or estrogen replacement therapy/
2.	Exp Estradiol/tu, th [Therapeutic use, therapy]
3.	Estrogens/tu, th [Therapeutic use, therapy]
4.	Epimestrol/tu, th [Therapeutic use, therapy]
5.	“Estrogens, conjugated (usp)”/tu, th [Therapeutic use, therapy]
6.	“Estrogens, esterified (usp)”/tu, th [Therapeutic use, therapy]
7.	Ethinyl estradiol/tu, th [Therapeutic use, therapy]
8.	Mestranol/tu, th [Therapeutic use, therapy]
9.	Exp quinestrol/tu, th [Therapeutic use, therapy]
10.	Progesterone/tu, th [Therapeutic use, therapy]
11.	Algestone/tu, th [Therapeutic use, therapy]
12.	Hydroxyprogesterones/tu, th [Therapeutic use, therapy]
13.	Exp selective estrogen receptor modulators/tu, th [Therapeutic use, therapy]
14.	Exp Medroxyprogesterone/tu, th [Therapeutic use, therapy]
15.	Exp progestins/tu, th [Therapeutic use, therapy]
16.	((Hormone or estrogen or oestrogen) adj (replacement or substitution or therap*)).mp.
17.	((Hormone or estrogen or oestrogen) adj (replacement or substitution) adj therap*).mp.
18.	(Selective adj (estrogen or oestrogen) adj receptor adj modulator*).mp.
19.	SERM.mp.
20.	(Medroxyprogesterone adj acetate).mp.
21.	(Estradiol* or progesterone* or medroxyprogesterone* or gestagen).mp.
24.	or/1-23
25.	Renal insufficiency/ or renal insufficiency, chronic/ or kidney failure, chronic/
26.	Exp dialysis/
27.	Renal replacement therapy/ or renal dialysis/ or hemodiafiltration/ or hemodialysis, home/ or peritoneal dialysis/ or peritoneal dialysis, continuous ambulatory/
28.	Kidney transplantation/
29.	((Renal or kidney) adj allograft).mp.
30.	((Renal or kidney) adj transplant*).mp.
31.	(Predialys* or pre-dialys*).mp.
32.	RRT.ti, kw.
33.	((Renal or kidney) adj replace* adj (therapy or treatment)).mp.
34.	(Peritoneal adj dialys*).mp.
35.	(hemodiafiltration or hemofiltration or haemodiafiltration or haemofiltration or hemodialys* or haemodialys* or dialys*).mp.
36.	(ESRD or ESKD).ti, kw.
37.	(End adj stage adj (renal or kidney) adj disease*).mp.
38.	((Renal or kidney) adj (insufficien* or failure or disease* or impair*)).mp.
39.	(Nephropathy or nephropathies).mp.
40.	(CKD or CRI).ti, kw.
41.	or/25-40
42.	Thromboembolism/
43.	“Intracranial embolism and thrombosis”/
44.	Carotid artery thrombosis/ or intracranial embolism/ or intracranial thrombosis/
45.	Embolism, paradoxical/ or venous thromboembolism/
46.	Thrombosis/
47.	Venous thrombosis/ or upper extremity deep vein thrombosis/
48.	"Embolism and Thrombosis"/ or Embolism/ or exp Pulmonary Embolism/
49.	(Thrombus or thrombi).mp.
50.	(Thrombosis or thromboemboli* or emboli*).ti, kw.
51.	(Venous adj thromboemboli*).ti, kw.
52.	((Arteriovenous or arterio-venous) adj (fistula or fistulae) adj (thrombosis or thrombus or clot* or emboli*)).mp.
53.	(AVF adj (thrombosis or thrombus or clot* or emboli*)).mp.
54.	or/42-53
55.	Exp Uterine Neoplasms/
56.	Exp Breast Neoplasms/
57.	Exp Genital Neoplasms, Female/
58.	Exp Ovarian Neoplasms/
59.	((Ovarian or ovary or breast or endometri* or uterus or uterine or genital) adj3 (neoplasm* or malignan* or cancer* or tumor* or tumour*)).mp.
60.	or/55-59
61.	(54 or 60) and 24 and 41
62.	Remove duplicates from 61

Description: MEDLINE key word and text word search strategy for Search #2

### Identification of study articles

A calibration exercise between the two reviewers will identify systematic discrepancies in abstract classification. Each reviewer, using the eligibility criteria of this study, will independently screen a total of 100 abstracts for inclusion or exclusion. A list of disagreements will then be constructed to identify the abstracts that resulted in discrepant results (inclusion vs. exclusion). The reviewers will discuss each reference that resulted in a disagreement, in order to determine potential rectifiable areas of interpretation of eligibility and classification. This process may lead to a reduction in disagreements within the article identification stage of the systematic review. Once calibration is complete, each reviewer will independently evaluate each article from the literature search for eligibility. This will be competed in a two-step process. The first step will involve a complete review of all identified abstracts. Those abstracts that are deemed eligible or have uncertain eligibility, as per the above eligibility criteria, will be selected for a full-text review. The second step will involve an independent review of each study selected for full-text review by the same reviewers. Article inclusion or exclusion will once again be determined by the above eligibility criteria. Each reviewer’s eligibility assessment of an article at both stages (abstract and full-text review) will be recorded and quantification of agreement between reviewers will be calculated using the Kappa statistic. Any disagreement between reviewers will be resolved by consensus at each stage of the screening process. If a consensus is not achieved, a third independent reviewer will serve as a final adjudicator.

### Data extraction

Two independent reviewers will extract relevant data from each eligible study using a standardized data extraction form. Results of data extraction will be compared between reviewers, and any discrepancy in data extraction will be resolved by consensus. The data will be organized and managed in Endnote (version X7, Thomson Reuters, New York, NY), Excel (version 2011, Microsoft Corporation, Redmond, WA), and Covidence (Cochrane Technology, Melbourne, Victoria, AU).

### Data items

Data items will include study identifiers (authors, location, year of publication), study design (sample size, inclusion and exclusion criteria, type of control, type of hormone therapy, as well as route, dose, timing of initiation, and duration of therapy), participant characteristics (age, comorbidities [CKD, diabetes mellitus, kidney transplant, previous VTE, known clotting disorders], family history of malignancy or clotting disorders, relation to menopause [perimenopausal vs. menopausal vs. primary ovarian insufficiency], type of menopause [natural vs. toxic vs. surgical], presence or absence of the uterus), and outcome data (loss of kidney function [change in creatinine clearance per 24 h urine collection or Cockcroft-Gault equation, change in estimated glomerular filtration rate per MDRD or CKD-EPI equations, inulin clearance or DTPA testing], presence or progression of albuminuria [change in urinary albumin-creatinine or protein-creatinine ratio or 24 h urine albumin or protein excretion, progression from normoalbuminuria to moderately increased albuminuria to severely increased albuminuria, progression to nephrotic range proteinuria], progression to ESKD [initiation of dialysis or receipt of kidney transplant], presence of hypertension [blood pressure >140/90, or as defined by authors], presence of breast, ovarian, or endometrial malignancy [in CKD population], development of VTE [in CKD population]).

### Risk of bias assessment

Quality and risk of bias in non-randomized studies will be assessed by each reviewer, using the Newcastle-Ottawa Scale [38]. The Cochrane risk of bias tool will be utilized by each reviewer to determine the quality and risk of bias of all randomized controlled trials [39]. Each trial will be further assessed with a Jadad score [40].

### Data synthesis and analysis

All studies resultant from the search described above will be presented using the PRISMA-P flow chart [41] including the initial number of studies, excluded studies, as well as reasons for exclusion. Data from the studies deemed eligible for the systematic review and meta-analysis will be summarized and quantitatively presented. The results of the systematic review will first be analyzed using descriptive statistics. Outcome measures will be presented as meta-analysis of RCTs and cohort studies separately, using random effects models. Measures of effect for dichotomous outcomes will be reported as risk ratios. Measures of effect for continuous outcomes, however, will be reported using weighted mean differences where common measurement instruments are used, or standardized mean differences if similar outcomes are presented using different measurement instruments between studies. If there are greater than or equal to two studies included in the analysis, methodologic and statistical heterogeneity (using the Cochrane *Q* statistic and the *I* ^2^ statistic) will be assessed to determine whether it is appropriate to report pooled measures of effect from meta-analysis. If there is any statistically significant heterogeneity, or if considerable methodological heterogeneity is noted by the reviewers, a univariable meta-regression and stratified analysis will be conducted to explore the potential sources of heterogeneity. The effects of the following variables on risk estimates will be assessed: (1) population-related (age, comorbidities, chronic kidney disease, relation to menopause [perimenopausal vs. postmenopausal], type of menopause, presence or absence of uterus), (2) intervention-related (type of treatment [postmenopausal HT vs. SERM], route, duration and dose), and (3) study design-related (blinding, allocation concealment, loss to follow-up in RCTs, and adjustment for prognostic factors and immortal time in cohort studies). Potential publication bias will be assessed via visual inspection of funnel plots, as well as Begg’s rank correlation test for asymmetry. All analyses will be completed using STATA (version 12, StataCorp LP, College Station, TX).

### Sensitivity analysis

Planned sensitivity analyses include determining if effects of postmenopausal HT differed by route of administration (i.e., oral vs. non-oral), with or without a concomitant progestin, or perimenopausal vs. postmenopausal status. In addition, we will perform sensitivity analyses with only low risk of bias studies as well as with only high risk of bias studies to determine what specific factors may be influencing the results.

### Limitations

Although this protocol is designed to minimize bias, there will be limitations of this systematic review. Firstly, because prescribing patterns of postmenopausal HT and SERMs are so diverse, the exposure variable is expected to be widely divergent. This may be with respect to the type, formulation, dosage, route, timing of initiation, and duration of treatment. Restriction of the search to a particular formulation or timing of initiation/duration, however, would likely lead to a paucity of data. We have prospectively planned to stratify analyses and meta-regression based on these aforementioned factors to explore any potential differences in outcome. Secondly, reported measures of kidney outcomes are not standardized and may be reported in different formats between studies. Thus, pooled analyses will be conducted to accommodate for the multiplicity of outcome measurements.

### Dissemination of results

The findings of this systematic review will be disseminated broadly by presenting the findings at scientific conferences, publishing the findings in a peer-reviewed journal, as well as publishing a summary of the data in newsletters and websites of invested organizations, decision-makers, and key stakeholders.

## Discussion

At present, it is unclear at this time as to how the administration of postmenopausal HT and SERMs affects the kidney health outcomes of postmenopausal women. This systematic review and meta-analysis is designed to assess these effects, as well as the risks of adverse outcomes of these treatments in women with preexisting CKD.

This systematic review has the potential for important clinical implications. With the aging population and the global rise in prevalence of CKD, the clinical scenario of optimizing care of the postmenopausal woman with or at risk of CKD will become increasingly common, highlighting the importance of this issue.

## Additional file

Additional file 1: PRISMA-P 2015 Checklist. Preferred Reporting Items for Systematic Reviews and Meta-Analyses for Protocols (PRISMA-P) checklist for development of protocol for systematic review and meta-analysis. (PDF 166 kb)

## Abbreviations

CKD: Chronic kidney disease
ESKD: End-stage kidney disease
HT: Hormone therapy
RCT: Randomized controlled trial
SERM: Selective estrogen receptor modulator
VMS: Vasomotor symptoms
VTE: Venous thromboembolism
